# Numerical Simulation of Nonperiodic Rail Operation Diagram Characteristics

**DOI:** 10.1155/2014/194975

**Published:** 2014-11-11

**Authors:** Yongsheng Qian, Bingbing Wang, Junwei Zeng, Xin Wang

**Affiliations:** ^1^School of Traffic and Transportation, Lanzhou Jiaotong University, Lanzhou 730070, China; ^2^Signal & Communication Research Institute, China Academy of Railway Sciences, Beijing 100081, China

## Abstract

This paper succeeded in utilizing cellular automata (CA) model to simulate the process of the train operation under the four-aspect color light system and getting the nonperiodic diagram of the mixed passenger and freight tracks. Generally speaking, the concerned models could simulate well the situation of wagon in preventing trains from colliding when parking and restarting and of the real-time changes the situation of train speeds and displacement and get hold of the current train states in their departures and arrivals. Finally the model gets the train diagram that simulates the train operation in different ratios of the van and analyzes some parameter characters in the process of train running, such as time, speed, through capacity, interval departing time, and departing numbers.

## 1. Introduction

With the rapid economic development in China, the higher requirements have been proposed for the operational efficiency of passenger and freight transports, as well as the human services. The train operation diagram is the basic file for organizing the train operation and the comprehensive plans of rail transport, playing a very important role in the organization of the entire rail transport system. The quality of the train operation diagram has great significance on improving the transport efficiency, accelerating the turnover rate and the delivery of passengers and freights, improving the usage of railway technology and equipment and meeting the needs of the market, and ensuring the safety accordingly. As for the railway running control system, the automatic block signalling has been widely utilized up to date. The automatic block signalling is a block system that consists of a series of signals that divide a railway line into a series of blocks and then functions to control the movement of trains between them through automatic signals. The train running state is normally controlled by a signalling system set in the course of its operation, and many different color light signalling systems have been used in the automatic block signalling. Namely, they are two-aspect color light, three-aspect color light, and four-aspect color light for the display format of automatic block signalling. Among them, the four-aspect color light system stage plays a dominant role in the automatic block signalling system. Under this signalling system, it presents four kinds of signals: red, yellow, yellow plus green, and green. If one of the blocking sections has been occupied by a train, the red signal will be on, indicating that this specific section is being occupied; if the section is free, the other signals will be on accordingly. In order to increase the train operation density in China, it is important to calculate the railway carrying capacity under the four-aspect color light automatic block signalling. The traditional calculation methods for the railway carrying capacity are the graphic method, the deduction coefficient method, and the average minimum train spacing interval law, respectively. All those methods are static algorithm and the empirical values are often introduced in, which, as a result, are likely to result in the lower accuracy.

On the aspect of trains diagram optimization, many experts have made plenty of research work on the train operation schemes [1–12]. Particularly, Meester and Muns studied the distribution of perturbation motion by “Phase-up” method as the prime condition; meanwhile, they used the probability theory to induce the condition of train's initial late [13]; Delorme et al. created the model of train's late speared; then, they used this method to study the characteristics of train diagrams and train speared [14]. In China, the railway system is under the heaviest task all over the world. There are also many achievements on train diagrams [15–20] and the methods of transportation model have been established already [21–27]. Focusing on the research field of the traffic flow, some Chinese experts have also attained several achievements of the CA model in both the theoretic research and practical application. Li et al. who had applied the NaSch model for the purpose of an analysis of train tracking and railway traffic flow for the first time proposed a CA model for simulating the railway traffic system. Two years later, Ning et al. [28] established a CA model to analyze and explore the space-time diagram of the railway traffic flow and the trajectories of the train movement. At present, the model for simulating the railway traffic system can be roughly divided into two classes: one for the moving block system and the other for the fixed block system. For the moving block system, more and more models for simulating the railway traffic system based on the fixed block system were proposed currently. Zhou et al. [29] simulated the traffic phenomenon of the delay propagation in a moving-like block system. Xun et al. [30] applied CA model to simulate the train running state as well as the traffic phenomenon of the delay propagation in the rail network. Fu et al. [31] proposed a CA model to simulate the tracking operation of trains in Beijing Subway Line 2. Li et al. established some sound rules to control the running process of a train and presented a new CA model with the consideration of the mixed trains and the distance between the adjacent stations to study the moving block system [32–34].

Unfortunately, all the above-mentioned studies on railway systems had not yet taken the passenger/freight ratio into consideration. In this work, the CA model of four-show fixed block system in the background of separated passenger and freight line is established in order to simulate the running process of trains and the influences of the different proportions of the passenger/freight on running processes are also discussed herewith. With this model, we simulated the train running state in the four-show fixed block system considering the intermediate stops as well as line maintenance nonperiod and obtained the simulating diagrams of different passenger/freight train ratios. Then, we numerically analyzed characteristics such as operation time, speed, capacity, spacing, and number.

## 2. Construction and Analysis of the CA Model

**Figure 1:**
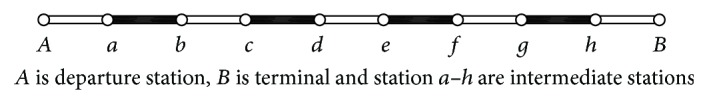
Rail line diagram.

The line examined in this paper is shown in Figure 1. We assume that the rail line *AB* is divided into *L* grids with equal length *l*; each cell is either empty or occupied by a train. Stations *A* and *B* as well as the intermediate station occupy a block subsection, respectively; each block subsection contains integer grids; namely, the length of the subsection is the integer multiple of *l*; the interval distance of any two of the stations contains integer block subsections; namely, the station spacing is also the integer multiple of *l*. Let the train speeds be an integer between 0 and *V*_*g*_, where *V*_*g*_ is the maximum allowable speed of the trains. Divide the analog line into a number of block subsections; each subsection contains a number of cells. Let the train run from left to right, and set the first signal light at the far left end of the rail line.

### 2.1. Define the Speed Limit Function

#### 2.1.1. Green-Yellow Light Speed Limit Function

If the signal light in front of the train is green-yellow, the train's speed should be less than or equal to the green-yellow speed limit function *V*_*gy*_(*s*), while *V*_*gy*_(*s*) should meet

(1) $$\begin{array}{c} V_{gy}{\left(s\right)}^{2}-V_{g}^{2}=2as,\;V_{gy}\left(s\right)\leq V_{g}, \end{array}$$

where *s* is the distance between the train and the front signal light, *a* is the train's acceleration, *V*_*gy*_(*s*) is the limit speed of green-yellow, *V*_*g*_ is the maximum allowable speed of the train when light turns green, and *V*_*gy*_ is the yellow speed limit. So we can get

(2) $$\begin{array}{c} V_{gy}\left(s\right)=\mathrm{int}\left[\min\left(\text{sqrt}\left(2as+V_{gy}^{2}\right),V_{g}\right)\right], \end{array}$$

where int⁡ stands for the rounding operation, min⁡ stands for the minimal value, and sqrt stands for the square root.

#### 2.1.2. Yellow Light Speed Limit Function

If the signal light in front of the train is yellow, the train speed should be less than or equal to the yellow speed limit function *V*_*y*_(*s*), while *V*_*y*_(*s*) should meet

(3) $$\begin{array}{c} V_{y}{\left(s\right)}^{2}-V_{y}^{2}=2as,\;V_{y}\left(s\right)\leq V_{gy}, \end{array}$$

where *s* is the distance between the train and the front signal light, *a* is the train's acceleration, *V*_*y*_(*s*) is the limit speed of yellow, *V*_*gy*_ is the maximum allowable speed of the train when light turns green-yellow, and *V*_*y*_ is the yellow speed limit. So we can get

(4) $$\begin{array}{c} V_{y}\left(s\right)=\mathrm{int}\left[\min\left(\text{sqrt}\left(2as+V_{y}^{2}\right),V_{gy}\right)\right]. \end{array}$$

#### 2.1.3. Red Light Speed Limit Function

If the signal light in front of the train is red, the train should stop. So we can get

(5) $$\begin{array}{c} V_{r}\left(s\right)=\mathrm{int}\left[\min\left(\text{sqrt}\left(2as\right),V_{y}\right)\right], \end{array}$$

where *s* is the distance between the train and the front signal light, *a* is the train's acceleration, and *V*_*r*_(*s*) is the limit speed of red.

#### 2.1.4. Train Passing the Station Speed Limit Function

If the light in front of the train shows the signal of passing the station, the speed of the train must be less than the station speed limit *V*_*z*_, when passing through the station through the home signal, and the station speed limit *V*_*tg*_(*s*) is

(6) $$\begin{array}{c} V_{tg}\left(s\right)=\mathrm{int}\left[\min\left(\text{sqrt}\left(2as+V_{z}^{2}\right),V_{g}\right)\right], \end{array}$$

where *s* is the distance between the train and the front signal light, *a* is the train's acceleration, *V*_*tg*_(*s*) is the limit speed of passing the station, and *V*_*z*_ is the limit speed of station.

#### 2.1.5. Train Entering and Stopping Speed Limit Function

Because the situation of entering and stopping is similar with that of the red light speed limit function, the speed limit functions of the two are the same:

(7) $$\begin{array}{c} V_{t}\left(s\right)=\mathrm{int}\left[\min\left(\text{sqrt}\left(2as\right),V_{y}\right)\right], \end{array}$$

where *V*_*t*_(*s*) is the train speed when entering and stopping.

### 2.2. Update Rules

#### 2.2.1. Speed Update

The speed of each vehicle according to the rules of Table 1 to update is as follows.

#### 2.2.2. Location Update

One has the following:

 
*X*_*n*_ = *X*_*n*_ + *V*_*n*_;
 
*X*_*n*_ is the location of train *n*.

#### 2.2.3. Color Update

One has the following:

 if *B*(*k*) = 1
 color(*k*) = “red”;
 else if *B*(*k* + 1) = 1
 color(*k*) = “yellow”;
 else if *B*(*k* + 2) = 1
 color(*k*) = “green-yellow”;
 else
 color(*k*) = “green”;
 end,

where *B*(*k*) represents the state of block subsection *k*, 1 is for the situation having train, and 0 is for the situation without train; color(*k*) represents the signal light color of the block subsection *k*.

Furthermore, the factor of the intermediate stops with unlimited capacity is also taken into account; that is, if the train needs to enter and stop, there are enough arrival and departure tracks for it. If the train that is getting nearer to the station needs to enter and stop, the train can directly enter the station if the conditions are met or else the train will have to stop in front of the station until the conditions are met; if the train only needs to go through the station, it can directly pass by the station. In addition, we also take the electric overhaul (every 20 hours) and maintenance (every 35 hours) time into consideration in this work.

## 3. Numerical Simulation and Analysis

### 3.1. Initialization of the Parameters

There are six stations in the simulation system, where the first station is the departure station and the last one is the terminal station. Others are intermediate overtaking stations, and the intermediate stations have infinite arrival and departure tracks; that is, the station capacity is infinite.

We assume that the length of the block subsection is 800 cells, the station spacing is 20 km, the total length of the line is 100 km, and the length of the train is 600 cells; the departure interval Tint is 7 min; there will be an electric overhaul every 20 hours and a maintenance every 35 hours, and every station will set a 120 min overhaul period. When the train is running through the terminal station, the train has pulled out of the analog system and the status of the train is no longer considered. The total number of the simulation steps is 259,200, namely, 72 hours. Trains in the departure station are allowed to depart in accordance with the time interval and safety conditions; trains in the intermediate stations can be allowed to depart as long as meeting the security conditions to start; if the station is in the maintenance period, trains cannot be allowed to depart. The symbols are defined as follows:

 L = 100000 cells, 1 cell = 1 m—the length of the line;
 
*V*_max⁡_^*p*^ = 35 cells/s = 126 km/h—the max speed of the passenger train;
 
*V*_max⁡_^*f*^ = 25 cells/s = 90 km/h—the max speed of the freight train;
 
*V*_*gy*_^*p*^ = 28 cells/s—the yellow-green light speed limit for the passenger train;
 
*V*_*gy*_^*f*^ = 20 cells/s—the yellow-green light speed limit for the freight train;
 
*V*_*y*_^*p*^ = 21 cells/s—the yellow light speed limit for the passenger train;
 
*V*_*y*_^*f*^ = 15 cells/s—the yellow light speed limit for the freight train;
 
*a* = 1 cell/s^2^ = 1 m/s^2^—the passenger and freight train's acceleration;
 
*b* = 1 cell/s^2^ = 1 m/s^2^—the passenger and freight train's deceleration.

### 3.2. Simulation Results and Analysis

**Figure 2:**
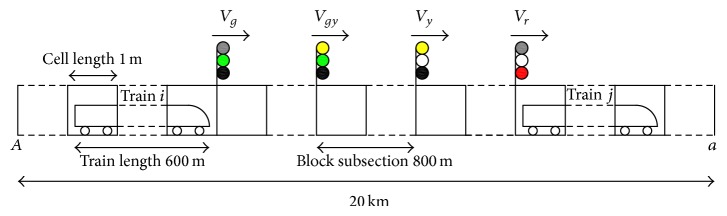
The operating condition of four-aspect colour light system.

Figure 2 shows the analog space-time diagram when the passenger/freight ratio is 4 : 1, in which the abscissa represents time, due to the large amount of output data, so 1 s in the figure represents the actual 5 s; the ordinate represents space. The horizontal lines in the figure indicate the stations; lines with small slope are the running lines of freight trains and lines with larger slope are the running lines of passenger trains.

**Figure 3:**
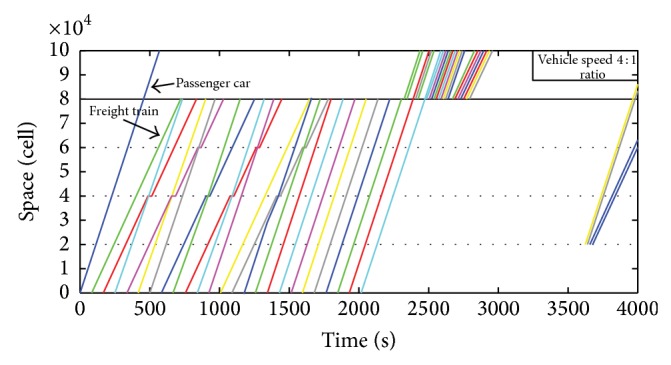
Operation diagram when the passenger/freight ratio is 4 : 1.

Figure 3 shows that a passenger train departing at time 0 from the departure station will directly go through the system because the line is train-free and is not in the maintenance period at this time. The third and fourth trains issued from the departure station are freight and passenger trains, respectively. It can be seen from the figure that the freight train departed before the passenger train; after passing the second station, the passenger train has caught up with and is following the freight train; at the third station, the freight train stops, and the passenger train overtakes it; when the passenger train travels out of the third station and the safety condition is met, the freight train will start to move on. When the passenger train travels into the fifth station, the station is in the maintenance period and it cannot pass, so the train stops at the station waiting for the overhaul being completed; all subsequent trains will also have to wait in the station until the maintenance period is finished. When the maintenance is completed, the station will take the centralized departure principles (passenger trains first; first come, first go) to give off all the detained trains as soon as possible. From the time of 2100 s when the maintenance is completed, the station begins to let the trains depart following the principles, until all the trains left.

**Figure 4:**
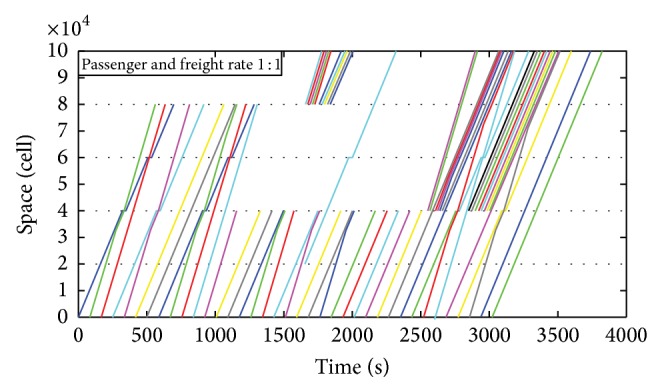
Operation diagram when the passenger/freight ratio is 1 : 1.

Figure 4 is the operation diagram when the passenger/freight ratio is 1 : 1. It can be seen from Figure 4 that due to the fact that the third station is in maintenance period all trains in the station have to wait until the end of the maintenance, which makes the road section between the third and the fifth stations be idle; after the maintenance period, the third station will take the centralized departure, which will make the road section be busy and will enhance the running load. So it is significantly meaningful to arrange the maintenance period and the departure principle reasonably.

Figures 3 and 4 show that, in the situation of mixed departure, all the trains do not have to stop in the second intermediate station, the reason for which is that the departure time interval is long enough, the freight train has entered the second section in this time interval, and it is impossible for the following passenger trains to catch up in the first section; in the second section, the passenger trains will catch up with the freight trains and then follow the latter until the third station, where the freight trains will stop to allow the passenger trains overtaking; then, the freight trains will move on.

**Figure 5:**
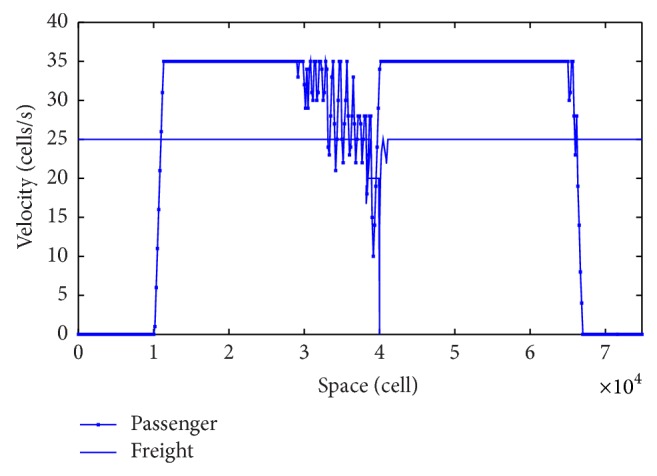
Space comparison chart between the passenger and freight trains.

Figure 5 is the space comparison chart between the passenger and freight trains, in which the front one is the freight train and the following one is the passenger train. It can be seen from Figure 5 that, after departure, the freight train will travel at the maximum speed; when it reaches the 10000 cells, the passenger train departed; when the passenger train reaches 30000 cells, its speed fluctuates continuously and tends to decelerate, while the speed of the freight train remains unchanged, indicating the state of steadily car-following; when close to 40000 cells, the speed of the freight train continues to reduce and finally becomes zero at 40000 cells, indicating that the freight train stops at the third station; while the passenger train is passing the third station, the speed drops to the minimum of 10 cells/s and then continues to accelerate to its maximum speed of 35 cells/s; and when it travels to 65000 cells, the speed of the passenger train will have two fluctuations and will reduce to 0, which is because of the maintenance period of the station.

**Figure 6:**
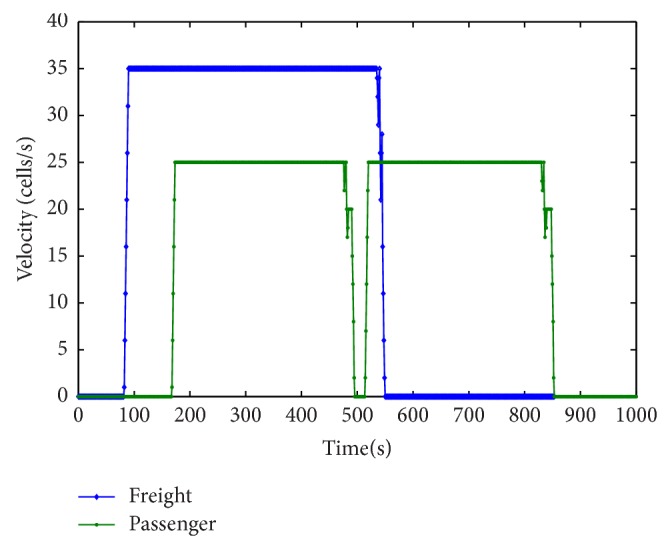
Time-speed comparison chart between the passenger and freight trains.

Figure 6 is the time-speed comparison chart between the passenger and freight trains. We can see from the figure that the first passenger train departed at 84 s from the departure station, and it will be pulled out of the system after about 470 s (actual 2350 s) with its maximum speed. A freight train departed at 160 s from the departure station, after about 335 s; the train will stop for short term to let the following passenger train go first, and then it will start running after about 320 s until it exits the system.

## 4. Conclusions 

In this paper, we proposed the cellular automata model for four-show fixed block system and simulated the train operation states considering the multi-intermediate stations as well as line maintenance period. Simulation results show that, in specific simulation environment, for different proportions of the train, the passing ability almost remains unchanged, which is because of the intermediate stations providing conditions for avoiding and parking and shortening delays. The results of this work can provide theoretical foundations, policy measures, and decision recommendations for the rail transport organizations and have a certain degree of theoretical and practical means for the enrichment and development of the theory and the practice of China's railway.

## Figures and Tables

**Table 1:** Speed update rules.

Train type	Color of signal	
	Green	Green-yellow
Passenger train	*V*_*n*_ = min⁡(*V*_*n*_ + *a*, *V*_max⁡_^*p*^)	*V*_*n*_ = min⁡(*V*_*n*_ + *a*, *V*_*gy*_(*s*))
Freight train	*V*_*n*_ = min⁡(*V*_*n*_ + *a*, *V*_max⁡_^*f*^)	*V*_*n*_ = min⁡(*V*_*n*_ + *a*, *V*_max⁡_^*f*^)

Train type	Color of signal	
	Yellow	Red

Passenger train	*V*_*n*_ = min⁡(*V*_*n*_ + *a*, *V*_*y*_(*s*))	*V*_*n*_ = min⁡(*V*_*n*_ + *a*, *V*_*r*_(*s*))
Freight train	*V*_*n*_ = min⁡(*V*_*n*_ + *a*, *V*_*y*_(*s*))	*V*_*n*_ = min⁡(*V*_*n*_ + *a*, *V*_*r*_(*s*))
